# Reduction of Arm Lymphedema Using Manual Lymphatic Therapy (Godoy Method)

**DOI:** 10.7759/cureus.28374

**Published:** 2022-08-25

**Authors:** Jose Maria Pereira de Godoy, Lívia Maria Pereira de Godoy, Henrique Jose Pereira de Godoy, Maria de Fatima Guerreiro Godoy

**Affiliations:** 1 Cardiology and Cardiovascular Surgery, Medicine School of Sao Jose do Rio Preto, São José do Rio Preto, BRA; 2 Angiology and Vascular Surgery, Clinica Godoy, São José do Rio Preto, BRA; 3 Dermatology, Instituto Lauro Souza de Lima-Bauru-Brazil, Bauru, BRA; 4 Dermatology, Clinica Godoy, São José do Rio Preto, BRA; 5 General Surgery, Medicine School of São José do Rio Preto-FAMERP, São José do Rio Preto, BRA; 6 General Practice, Clínica Godoy, São José do Rio Preto, BRA; 7 Physical Rehabilitation, Medicine School of São José do Rio Preto, São José do Rio Preto, BRA; 8 Rehabilitation, Clinica Godoy, São José do Rio Preto, BRA

**Keywords:** evaluation, therapy, manual lymphatic drainage, breast cancer, lymphedema

## Abstract

Background: The manual lymphatic drainage (MLD) technique used during the early stages following surgical treatment of breast cancer can help prevent the progression of clinical lymphedema.

Objective: The objective of this study was to evaluate the effectiveness of manual lymphatic therapy (MLT) (Godoy method) in reducing the development of lymphedema immediately after breast cancer treatment.

Method: A randomized, blind, crossover, clinical trial was conducted involving 66 women with breast cancer-related lymphedema (BCRL), who underwent one hour of manual physical therapy and one hour of the control procedure. To evaluate the volume before and after the application of the MLT technique, volumetry, a water displacement technique was used. For statistical analysis, the paired t-test with 5% alpha error by* *Stats Direct 3(StatsDirect Ltd, Wirral, UK) was used.

Results: A significant reduction in the volume of the limb was found in all patients (p-value = 0.0001, paired t-test).

Conclusion: MLT is effective in reducing lymphedema after breast cancer treatment.

## Introduction

Manual lymphatic drainage (MLD) technique used during the early stages following surgical treatment for breast cancer can help prevent the progression of lymphedema. It can also provide additional benefits regarding the reduction in volume in cases of mild lymphedema, but not in cases of moderate to severe lymphedema when combined with complex decongestive therapy [1]. In controversy to this study, results of other research’s randomized clinical trials show that MLD does not significantly reduce or prevent lymphedema in patients following surgical treatment for breast cancer [2-3].

The incidence of breast cancer-related lymphedema (BCRL) was shown to be 24.8%-90.4% as per the literature. Several factors have been associated with lymphedema following conservative breast surgery, such as the body mass index (BMI), breast size, tumor size, tumor location, type of surgery, and adjuvant therapy [4].

Regarding treatment, MLD is the most consolidated form of therapy. After Leduc, the Vodder couple made some changes and developed their technique, which was the most widely disseminated technique for decades [5-6]. In recent years, Godoy and Godoy have developed novel concepts in lymphatic therapy, new technique of manual lymphatic therapy (MLT) with linear movements, adapted physiopathology for breast cancer, as well as a method of lymphatic cervical stimulation, which consisted of approximately 30 gentle surface movements of 0.5 cm in the cervical region for 15-20 min [7-11].

One of the considerable challenges regarding MLD is the existence of several “massage” techniques developed in different parts of the world called MLD, but no scientific studies have been conducted to evaluate the results, which compromises its credibility [3]. The Godoy has extensive scientific publications related to lymphatic therapy techniques [7-15]. The objective of this study was to evaluate the effectiveness of MLT (Godoy Method) in reducing the immediate development of lymphedema after breast cancer treatment.

## Materials and methods

Sample

Caucasian women with lymphedema who have undergone a prior mastectomy, tumorectomy, or quadrantectomy and axillary lymphadenectomy. A sample size of 66, and mean age was 61.1 years (range: 51-82 years). Evaluations and treatment were performed at the Clínica Godoy, Brazil.

Study design

A randomized, blind, crossover, clinical trial was conducted involving 66 women with BCRL, using clinical diagnostics, immediately after treatment, who underwent one hour of MLT (Godoy Method -- linear movements in cephalic channel and posterior channel) and one hour of the control procedure (rest). Volumetric analysis (water displacement) was performed before and after the treatment and differences were analyzed using the paired t-test.

Inclusion criteria

Patients with BCRL, clinical diagnosis with a limb volume difference of at least 200 mL compared to the contralateral upper limb.

Exclusion criteria

Primary lymphedema or other causes of edema detected during the patient history and physical examination.

Ethical considerations

This study received approval from the institutional review board of the São Jose do Rio Preto School of Medicine, São Jose do Rio Preto, São Paulo, Brazil#387.

Statistical analysis

To evaluate the volume before and after the application of the MLT technique by volumetry, a water displacement technique. For statistical analysis paired t-test with 5% alpha error by Stats Direct 3 was used. 

Development

Patients with a history of cancer treatment that resulted in the development of lymphedema sought care at the Clinica Godoy, Brazil. The patient history was taken, followed by a physical examination and complementary exams, such as volumetry (water displacement method, circumference measurements, and multi-segment bioimpedance analysis). After the diagnosis, the patient received clarifications regarding the study and agreed to participate. Volumetry was performed again immediately prior to MLT and immediately after the session. A control group was also formed by the patients themselves; the sequence (control evaluation or treatment) was determined by a coin toss. The Godoy Method of MLT was performed for one hour, alternating three drainage techniques: cephalic chain, posterior chain, and compression therapy. The technique consists of linear movements along the trajectories of the cephalic and posterior chains and manual compression therapy along the trajectories of the main collectors involved with axillary clearance.

## Results

**Figure 1:**
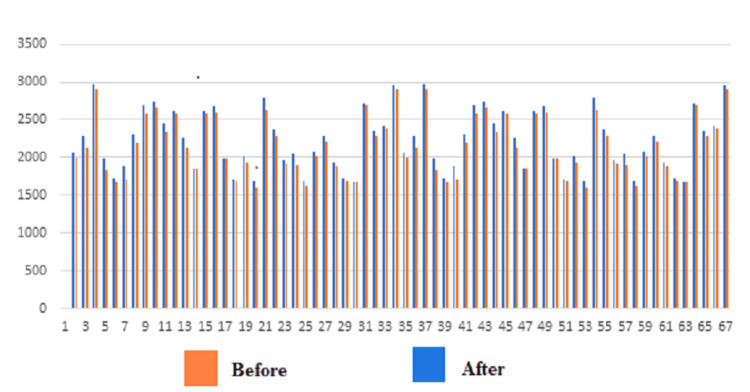
Volumes before and after MLT. MLT, manual lymphatic therapy

A significant reduction in the volume of the limb was found in all patients after treatment with MLT (Godoy Method) (p-value = 0.0001, paired t-test). Table 1 shows the data of the descriptive statistics analysis compared by volumetry before and after immediately after the one hour session of MLT and compared with the control procedure of this group patients following the rehabilitation treatment in Clinica Godoy. Figure 1 shows the volume (mL) before and after one hour of MLT (Godoy Method).

**Table 1:** Descriptive statistics of data before and after 1 h treatment (MLT) and comparison of analysis volume reduction control group. MLT, manual lymphatic therapy

Variables	Before	After	Volume reduction ( (mL)	Volume reduction/control group
Valid data	66	66	66	66
Mean	2229.30	2153.96	66.57	24.80
Standard deviation	393.091	388.71	30.37	20.56
Maximum	2972	2898	130	64
Median	2265	2125	69	19
Minimum	1682	1598	8	2

## Discussion

The present study shows that MLT is effective in reducing the volume of limbs with lymphedema after one hour of treatment. This is the first MLD technique in the literature to be studied as a monotherapy in the treatment of lymphedema [8, 10-15]. The evaluation of this study was conducted immediately after treatment. Thus, it is important to determine whether the results of monotherapy will be maintained and which strategy should be used as an effective technique in the treatment of lymphedema. This is a constant concern of the authors. Short- and long-term studies have been conducted evaluating the drainage technique developed [11-13] and assessments have been performed using lymphoscintigraphy [14-15].

We have observed that several massage techniques that have been called as lymphatic drainage techniques without scientific evaluation and, when used in the treatment of lymphedema, can cause serious harm to these patients [1-4]. It is paramount for the treatment of a disease to have a scientific basis. Another mistake is not to distinguish what is lymphatic drainage and what is massage, which is a completely different thing. Drainage involves the use of a specific method performed correctly that does not cause additional harm to the lymphatic system. These criteria are fundamental to the scientific credibility of the technique. Therefore, for every scientific evaluation of a method, the author needs to have profound knowledge of the technique.

Several meta-analyses have shown that MLD for the treatment of BCRL is not effective in more severe cases. Indeed, a large number of sessions would be needed in such cases, which is unviable from a practical standpoint. In a 30-month study, a healthcare professional spent half of the day dedicated to such cases [13]. This was done in cases of lymphedema of the lower limbs. In another study, we evaluated each maneuver specifically (cephalic chain, posterior chain, and manual compression therapy) in the region of the vessels involved in surgery to determine whether these techniques are effective and whether one is superior to another [10].

Significantly, better results are achieved when braces and elastic sleeves are used immediately after the MLD session [16]. Thus, the combination of techniques is necessary. This improvement demonstrates a positive synergic effect. However, some combinations do not achieve the same result as monotherapy, in which case, we have a negative synergic effect. Therefore, the combination of therapies needs to be evaluated well in order to achieve the best results.

We have assessed combinations for the treatment of upper limb lymphedema for long periods [17-18] as well as the immediate effect of the combination of exercises and compression mechanisms [19-20] with the specific investigation of aggravating factors in these patients [21-22]. What we have observed is that the treatment of lymphedema in general and BCRL specifically requires a set of care procedures. The maintenance of the results is another important aspect. We have found that frequent control is necessary, along with the adaptation of a simpler form of daily control. Such aspects interfere with adherence to treatment. The intensive treatment of lymphedema by Godoy method is another important contribution, which involves a combination of therapies eight hours per day for five days and is a possible method to reduce the volume of the limb by approximately 50%. The ultimate goal is to achieve the clinical normalization or near normalization of the limb.

All therapeutic progress requires innovative scientific research with the creation of novel concepts and techniques. Currently evaluating the reversal of fibrosis in lower limb lymphedema and the initial results show a general change in the structure of the skin involving the entire extracellular matrix, type I and III collagen fibers, reticular fibers and epidermis as well as a novel cell type that is under analysis: telocytes. Thus, we will soon have confirmed what we have been divulging for years -- that it is possible to reverse fibrosis in the treatment of lymphedema [23-24]. The limitation of the study, we believe, was that it did not include patients of the same age, the same type of surgery and post-surgery treatment (radiotherapy, chemotherapy) and similar time that lymphedema developed and to compare volume reduction with TLM (Godoy method) with other MLD techniques.

The limitation of the study, we believe, was that it did not include patients of the same age, the same type of surgery and post-surgery treatment (radiotherapy, chemotherapy) and similar time that lymphedema developed and to compare volume reduction with TLM (Godoy method) with other MLD techniques.

## Conclusions

In this study, the effect of MLT (Godoy Method) on volume decreasing lymphedema was proven by the evaluation using volumetry. This suggests MLT is effective in decreasing the volume of lymphedema as it is a technique adapted to the pathophysiology. Further studies involving manual lymphatic therapy as a form of isolated therapy are needed to determine the effectiveness of the therapy in the treatment of lymphedema.
